# Virtual exam for Parkinson’s disease enables frequent and reliable remote measurements of motor function

**DOI:** 10.1038/s41746-022-00607-8

**Published:** 2022-05-23

**Authors:** Maximilien Burq, Erin Rainaldi, King Chung Ho, Chen Chen, Bastiaan R. Bloem, Luc J. W. Evers, Rick C. Helmich, Lance Myers, William J. Marks, Ritu Kapur

**Affiliations:** 1 Verily Life Sciences, South San Francisco, CA USA; 2 Radboud University Medical Center, Donders Institute for Brain, Cognition and Behaviour, Department of Neurology, Center of Expertise for Parkinson & Movement Disorders, Nijmegen, the Netherlands; 3 Radboud University, Institute for Computing and Information Sciences, Nijmegen, the Netherlands

**Keywords:** Biomarkers, Parkinson's disease

## Abstract

Sensor-based remote monitoring could help better track Parkinson’s disease (PD) progression, and measure patients’ response to putative disease-modifying therapeutic interventions. To be useful, the remotely-collected measurements should be valid, reliable, and sensitive to change, and people with PD must engage with the technology. We developed a smartwatch-based active assessment that enables unsupervised measurement of motor signs of PD. Participants with early-stage PD (*N* = 388, 64% men, average age 63) wore a smartwatch for a median of 390 days. Participants performed unsupervised motor tasks both in-clinic (once) and remotely (twice weekly for one year). Dropout rate was 5.4%. Median wear-time was 21.1 h/day, and 59% of per-protocol remote assessments were completed. Analytical validation was established for in-clinic measurements, which showed moderate-to-strong correlations with consensus MDS-UPDRS Part III ratings for rest tremor (⍴ = 0.70), bradykinesia (⍴ = −0.62), and gait (⍴ = −0.46). Test-retest reliability of remote measurements, aggregated monthly, was good-to-excellent (ICC = 0.75–0.96). Remote measurements were sensitive to the known effects of dopaminergic medication (on vs off Cohen’s d = 0.19–0.54). Of note, in-clinic assessments often did not reflect the patients’ typical status at home. This demonstrates the feasibility of smartwatch-based unsupervised active tests, and establishes the analytical validity of associated digital measurements. Weekly measurements provide a real-life distribution of disease severity, as it fluctuates longitudinally. Sensitivity to medication-induced change and improved reliability imply that these methods could help reduce sample sizes needed to demonstrate a response to therapeutic interventions or disease progression.

## Introduction

Parkinson’s disease (PD) affects 6 million people worldwide as of 2018—a number that is projected to grow to 12 million by 2040^1^. Treatments are being developed to slow down or even halt the progression of PD^2,3^. However, currently used endpoints (e.g., the MDS-UPDRS) exhibit high within-subject variability, and low test-retest reliability, which leads to inefficient clinical trials, and risks potentially missing relevant effects^4^. Compounding this challenge, clinic-based physical exams provide only a snapshot of PD signs, and may not adequately reflect a patient’s functioning at home^4,5^. Additionally, many people live far from major medical centers^6^, so access to clinical trials of new therapeutics becomes restricted to a limited portion of the Parkinson population^7,8^.

These challenges have motivated the search for digital endpoints using wearable sensors, which allow for objective, frequent, and ecologically valid measurements of motor functioning in the patient’s home environment. Sensor-based remote monitoring could also help increase representation for groups whose data have historically not been included in clinical trials^9,10^. Before such measurements can be used as endpoints in clinical trials to quantify disease progression, a careful evaluation of the clinical validity, reliability, and sensitivity to change is required^11,12^.

A substantial volume of research has demonstrated the feasibility of using sensors placed on various parts of the body to quantify motor signs of PD^13^. Results suggest that features extracted by digital signal processing can be correlated with clinical outcomes of interest, at least when tests are delivered in a controlled setting and assessments are supervised by a clinician^14–22^. Active assessments measure patients’ maximum capacity, and can be complementary to passive monitoring, which measures the expression of signs in real life. Though some studies have probed the feasibility of using wearable sensors or smartphones for remote, self-guided active assessments, long-term engagement - which is critical to study disease progression - has been an important challenge^23–25^. Studies focusing on passive monitoring of PD motor signs have generally not been able to capture a person’s intent to move, which is particularly relevant for signs of bradykinesia^26^. Moreover, most of the existing work has focused on comparing sensor-based measures to clinical ratings, with limited work systematically measuring the ability of the remote measures to detect the effects of dopaminergic medication. Finally, test-retest reliability and sensitivity to clinically meaningful change have rarely been reported, and generally not on a large scale.

The smartwatch-based Parkinson’s Disease Virtual Motor Exam (PD-VME) can be deployed to remotely measure the severity of tremor, bradykinesia, and gait impairment, via a self-guided active assessment^27^. Here, we evaluate the feasibility of use and quality of data collected by the system, and report on the reliability, validity, and sensitivity to change of a set of digital measures derived from the PD-VME during a multi-year deployment in the Personalized Parkinson Project (PPP)^27^.

## Results

Data were collected as part of the ongoing Personalized Parkinson Project (PPP), a prospective, longitudinal, single-center study (Clinical Trials NCT033648) of 520 people with early-stage Parkinson’s disease—diagnosed within the last 5 years^27^. Study participants wear a smartwatch (Verily Study Watch) for up to 23 h/day for the 3-year duration of the study, which passively collects raw sensor data from IMU, gyroscope, photoplethysmography, and skin conductance sensors.

Set 1 (*N* = 198 participants) was selected for video-based consensus scoring by matching age, gender, and MDS-UPDRS III score to be representative of the overall PPP study. Two assessors independently scored videos of the exams. When difficulties in rating MDS-UPDRS Part III tasks arose due to poor video quality, assessors provided scores only when confident in their assessment. MDS-UPDRS Part III consensus scores were computed as the median of the in-person rating and both video ratings.

Starting in May 2020, participants were offered the opportunity to enroll in a substudy, which asks them to perform an active assessment (Parkinson’s Disease Virtual Motor Exam, PD-VME) in the clinic and in remote, unsupervised settings. The PD-VME was deployed fully remotely, using digital instructions and an over-the-air firmware update to the watches of consented participants. A total of 370 participants enrolled in the substudy (Set 2).

**Fig. 1:**
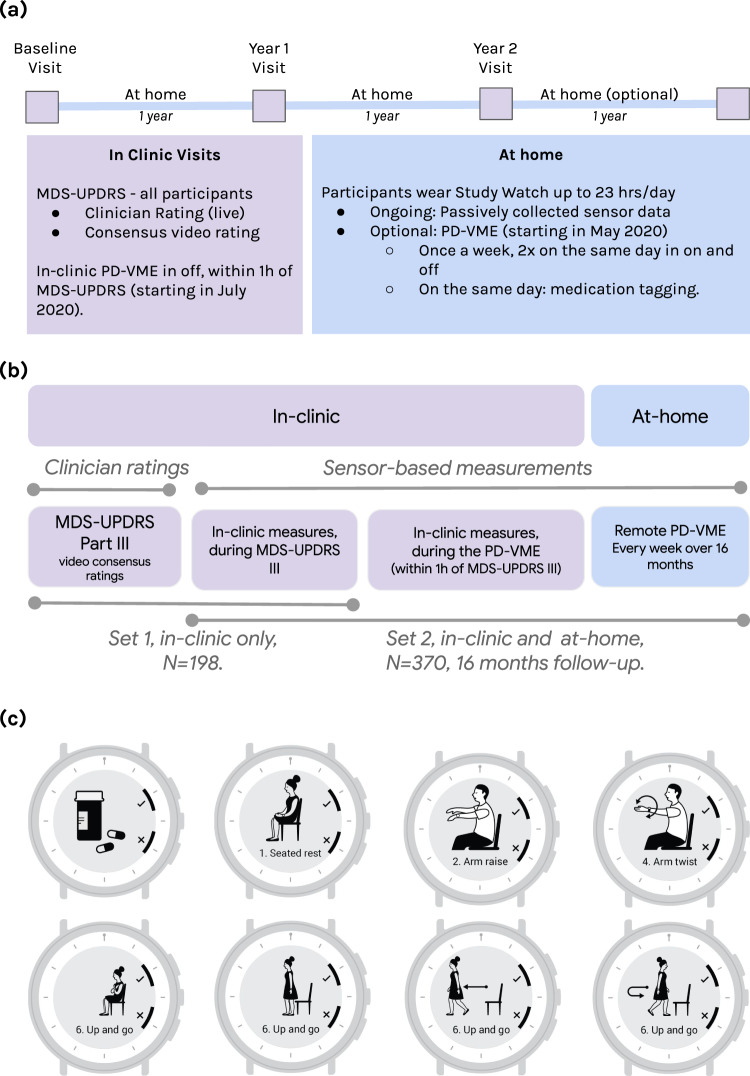
Study design and participant-facing interface. **a** Study design for the Personalized Parkinson’s Project. **b** Study schematic defining Sets 1 and 2 for the PD VME substudy. Set 1 consists of participants for whom consensus ratings of the MDS-UPDRS were available. Set 2 consists of participants who opted into the PD-VME substudy and performed at least one PD-VME. **c** User interface to prompt medication logging and PD-VME tasks. Seated rest, arm raise and arm twist (20 s duration), up-and-go (60 s duration).

The smartwatch guides participants through the series of structured motor tasks comprising the PD-VME. It also allows patients on symptomatic medication to log the timing of their medication intake. The study design and patient-facing UI of the PD-VME are summarized in Fig. 1.

Each week, participants were asked to perform the PD-VME twice on the same day, at two predefined times: first in the off state (selected as a time when they typically experienced their worst motor function), and then in the on-state (at a time when they typically experienced good motor function later in the day). Participants not taking medication were instructed to complete the PD-VME twice, one hour apart. The helpdesk at the site (Radboudumc) monitored wear-time and PD-VME completion and reached out to participants if more than three consecutive weekly assessments were missed.

Starting in July 2020, participants enrolled in the PD-VME substudy were asked to perform the PD-VME during their in-clinic visit (in the same manner as they did remotely), while the assessor observed its execution without providing feedback or any additional instructions. The in-clinic PD-VME is performed within 1 h after completion of the MDS-UPDRS part III off state exam, and before dopaminergic medication intake.

Demographic and clinical characteristics of the study population are presented in Table 1, for participants in Set 1 and Set 2. Distributions of the side on which the participants chose to wear the smartwatch are also included.

**Table 1:** Participant demographics and characteristics at their first clinic visit.

		Set 1 (consensus)^1^	Set 2 (PD-VME)^2^
Demographics			
N^3^		198	370
Age (years)	Mean (SD)	62.9 (8.9)	62.3 (8.8)
Gender	Men, *n* (%)	128 (64.6)	231 (62.4)
Side on which the watch was worn	Left, *n* (%)	182 (92.0)	342 (92.4)
Side on which the watch was worn	Most affected, *n* (%)	117 (59.1)	196 (53.0)
Disease at onset			
Time since diagnosis (years)	Mean (SD)	3.3 (1.4)	2.9 (1.4)
Disease severity			
MDS-UPDRS Part I	Mean (SD)	15.7 (8.3)	15.1 (7.7)
MDS-UPDRS Part II	Mean (SD)	9.6 (6.4)	8.4 (5.7)
MDS-UPDRS Part III (Off state)	Mean (SD)	37.6 (15.3)	33.8 (13.0)
Dyskinesia observed in clinic	Count	27	37
Hoehn and Yahr Scale	Mean (SD)	2.1 (0.6)	2.0 (0.5)
Montreal Cognitive Assessment (MOCA)	Mean(SD)	26.6 (2.8)	26.7 (2.5)
Medications			
Participants on any symptomatic medication	*n* (%)	185 (93%)	297 (80%)
Participants on fast-acting medication^4^	*n*, (%)	117 (59%)	202 (54%)

^1^Set 1 (consensus) includes all participants who had at least two independent video ratings and one in-clinic rating. ^2^Set 2 (PD-VME) includes all participants who had at least one PD-VME exam. ^3^180 participants were included in both sets, for a total of 388 unique participants. ^4^excludes dopamine agonists.

### Engagement

Median smartwatch wear time across all PPP participants (*N* = 520)^27,28^ was 22.1 h/day, with a median follow-up period of 390 days. Variations in follow-up duration are due largely to the *N* = 126 who have not completed the study at the time of publication, and loss-to-follow-up is only 5.4%. Reasons for participant drop-out are indicated in Supplementary Table 2. Participants in Set 2 completed 22,668 PD-VMEs, corresponding to 59% of per-protocol test sessions during the 70-week follow-up period (Supplementary Fig. 1). In the first week, 80% of participants had at least 1 PD-VME, and 40% had completed one PD-VME in week 52.

### Useability

Participants’ ability to perform the PD-VME was assessed during the in-clinic visit. Participants were able to complete the tasks in the exam (100% for tremor and upper-extremity bradykinesia and 98.5% for gait). Major protocol deviations were recorded as follows: participants did not place their hands on their lap during rest tremor tasks (8.2% of cases), participants performed the arm-twist using both arms (3.1% of cases), and participants either walked with their arms crossed across their chest (in 3.1% of cases) or sat down repeatedly (6.8% of cases) during the gait task. Detailed results are summarized in Supplementary Table 3.

### Rest tremor

Among three measurements that were considered for measuring tremor severity, lateral tremor acceleration measurement is presented here because it showed the strongest correlation to in-clinic MDS-UPDRS ratings, and the strongest ability to separate on state from off state measurements. Results for additional measures are included in Supplementary Table 4.

**Fig. 2:**
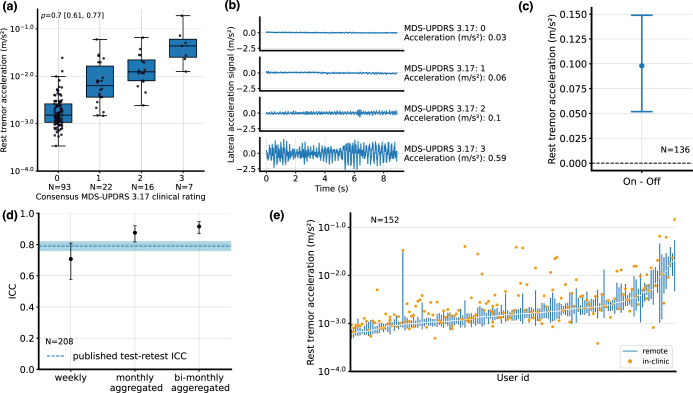
Rest tremor acceleration. **a** Lateral tremor acceleration (log scale), measured during the in-clinic examination, by rest tremor (MDS-UPDRS 3.17) consensus score. Center lines: median, boxes: first and third quartiles, whiskers: 1.5x inter-quartile range. **b** Illustrative examples of raw lateral acceleration signals for each score on the MDS-UPDRS 3.17. Measurement values, as computed by the PD-VME, are also indicated. **c** Difference between the remote measurements in on and off states, aggregated over PD-VMEs obtained during the first two months from each participant. Mean and 95% confidence intervals across participants are represented. **d** Intra-class correlation (ICC) between at-home measurements, for various durations of aggregation. Whiskers represent 95% confidence intervals. The dotted blue line represents the published test-retest ICC of 0.79 for the whole rest tremor UPDRS Part III subcomponent (all four extremities + lip & jaw)^44^. **e** Distribution of PD-VME tremor measurements (off state) obtained during the in-clinic PD-VME (orange dot, representing a single measurement) and remote PD-VMEs (blue bar, representing the 25th to 75th percentile of PD-VMEs within 90 days of the in-clinic PD-VME), sorted on the remote PD-VMEs.

The Spearman rank correlation between the median lateral acceleration during the rest tremor task and expert consensus rating of MDS-UPDRS task 3.17 was 0.70 [0.61, 0.77], *N* = 138 (Fig. 2a). For 56 participants, video quality was insufficient to ensure high confidence consensus ratings wrist acceleration signals intuitively map to the clinical observations during the MDS-UPDRS (Fig. 2b). Next, the sensitivity to on-off changes of the rest-tremor acceleration measurement was assessed (Fig. 2c). A small effect (Cohen’s d of 0.2) was observed comparing the on and off state. The mean difference in the measure was 0.10 [0.05, 0.1].

Test-retest reliability is reported in Fig. 2d, with intra-class correlation (ICC) of 0.71 [0.58–0.81] week-on-week (*N* = 208), and ICC of 0.90 [0.84–0.94] m s^−2^ for monthly averaged measures (N = 139).

Finally, the distribution of remote measurements compared to the sensor measurement during the in-clinic VME is shown in Fig. 2e. The in-clinic PD-VME measure was between the 25th and the 75th percentiles of the remote PD-VME measures for 41% of the participants.

Additional results for Postural tremor are included in Supplementary Table 5 and Supplementary Fig. 2.

### Upper-extremity bradykinesia

Among the four measurements that were considered for measuring upper-extremity bradykinesia severity, no single measure showed both strong correlation to in-clinic MDS-UPDRS ratings, and a strong ability to separate on from off state measurements. Therefore, results are included below for both the arm-twist amplitude, and the arm-twist rate.

**Fig. 3:**
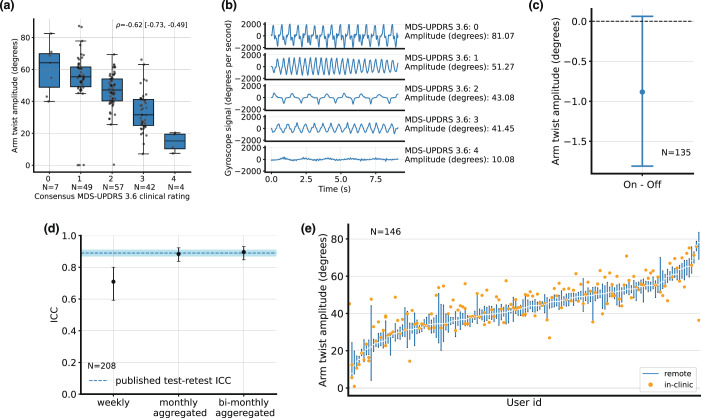
Upper extremity bradykinesia. **a** Arm twist amplitude measured during the in-clinic examination, by pronation-supination (MDS-UPDRS 3.6) consensus scores. Center lines: median, boxes: first and third quartiles, whiskers: 1.5x inter-quartile range. **b** Illustrative examples of raw gyroscope signals, along the x axis, for each score on the MDS-UPDRS 3.6. Measurement values, as computed by the PD-VME, are also indicated. **c** Difference between the remote measurements in on and off states, aggregated over PD-VMEs obtained during the first two months from each participant. Mean and 95% confidence intervals across participants are represented. **d** Intra-class correlation (ICC) between at-home measurements, for various durations of aggregation. Whiskers represent 95% confidence intervals. The dotted blue line represents the published test-retest ICC of 0.89 for the whole bradykinesia subcomponent of the UPDRS Part III^31^. **e** Distribution of PD-VME arm-twist measurements (off state) obtained during the in-clinic PD-VME (orange dot, representing a single measurement) and remote PD-VMEs (blue bar, representing the 25th to 75th percentile of PD-VMEs within 90 days of the in-clinic PD-VME), sorted on the remote PD-VMEs.

The highest correlation with expert consensus rating of MDS-UPDRS task 3.6 was observed for the arm twist amplitude measure, with ρ = −0.62 [−0.73, −0.49], *N* = 159 (Fig. 3a). However, the effect of medication state (Cohen’s d of −0.07) was very small (Fig. 3c)^29^. The mean on-off difference in the measure was −0.9 [0.0, −1.6] degrees. Test-retest ICC (Fig. 3d) was 0.71 [0.59–0.80] week-on-week (*N* = 208) and 0.89 [0.84–0.94] for monthly-averaged measures (*N* = 136). The in-clinic PD-VME measure was between the 25th and the 75th percentiles of the remote PD-VME measures for 45% of the participants.

The assessors observed during the in-clinic PD-VME exam that some patients mainly focussed on the speed of the arm-twist movement rather than the amplitude. Therefore, sensor-based measures of the rate of arm-twist and the combination of rate and amplitude were investigated as well. Correlations to the consensus MDS-UPDRS ratings of ρ = 0.06 [−0.25, +0.13] for arm-twist rate, and ρ = −0.42 [−0.55, −0.28] for the product of rate and amplitude were observed. Both metrics showed significant change in on and off: Cohen’s d of −0.22 and mean change of −0.16 [−0.13, −0.20] s^−1^ for arm-twist rate, and Cohen’s d of −0.26 and mean change of −8 [−6, −10] degrees/s for the combination. The full results are included in Supplementary Table 6.

### Arm swing during gait

Among the three measurements that were considered for measuring gait impairment, arm swing acceleration was selected. While it was not the best outcome measure across any of the criteria, it showed solid performance across all of them. Results for the measures that were not selected are included in Supplementary Table 7.

**Fig. 4:**
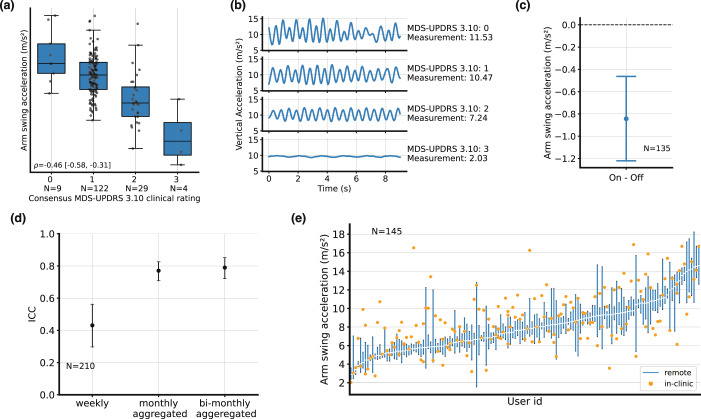
Gait. **a** Arm swing acceleration measured during the in-clinic examination, separated by gait (MDS-UPDRS 3.10) consensus scores. Center lines: median, boxes: first and third quartiles, whiskers: 1.5x inter-quartile range. **b** Illustrative examples of raw accelerometer signals, for each score on the MDS-UPDRS 3.10. Measurement values, as computed by the PD-VME, are also indicated. **c** Difference between the remote measurements in on and off states, aggregated over PD-VMEs obtained during the first two months from each participant. Mean and 95% confidence intervals across participants are represented. **d** Intra-class correlation (ICC) between at-home measurements, for various durations of aggregation. Whiskers represent 95% confidence intervals^44^. **e** Distribution of PD-VME gait measurements (off state) obtained during the in-clinic PD-VME (orange dot, representing a single measurement) and remote PD-VMEs (blue bar, representing the 25th to 75th percentile of PD-VMEs within 90 days of the in-clinic PD-VME), sorted on the at-home PD-VMEs.

The Spearman rank correlation between the arm swing acceleration during the gait task and expert consensus rating of MDS-UPDRS task 3.10 was ρ = −0.46 [−0.58, −0.31], *N* = 164 (Fig. 4a). A small effect (Cohen’s d of 0.44) was observed comparing the on and off state. The mean difference in the measure was −0.8 [−1.2, −0.5] m−s^−2^. Test-retest ICC (Fig. 4d) was 0.43 [0.30–0.56] week-on-week (*N* = 210), and 0.75 [0.66–0.84] for monthly-averaged measures (*N* = 139). The in-clinic PD-VME measure was between the 25th and the 75th percentiles of the remote PD-VME measures for 39% of the participants.

## Discussion

The data from this study suggest that people with PD engage with and are able to use the PD-VME, and that the quality of data collected is high enough to enable evaluation of the analytical validity, reliability, and sensitivity to change of digital measures built from this system.

A digital solution is only useful if people with PD engage with it regularly. We observed robust levels of engagement, both in terms of overall wear time (>21 h/day) and engagement with the active assessment, which was 59% over one year when assayed on a weekly basis. This is at the high end of reported values^23,24^, and this suggests that combining active assessments with passive monitoring on smartwatch form-factors have the potential to yield substantial quantities of high-quality data. For studies assessing longitudinal progression, our observations suggest that even higher engagement could be obtained by requiring a set of weekly unsupervised tests for a limited duration at baseline and again at the end of the follow-up period.

We showed a moderate-to-strong correlation between in-clinic MDS-UPDRS Part III measurements and consensus clinical ratings for rest tremor, bradykinesia, and arm swing during gait, which provided analytical validation of the individual measurements. These results are on par with similar published analyses of wrist-worn sensors^30–33^ and demonstrate the ability of the PD-VME to provide metrics that map to the observations of an expert clinician. While the moderate-to-strong correlations with MDS-UPDRS scores establish that the measurements are working as intended, engineering for perfect correlation simply recreates an imperfect scoring system, and washes out the potential for increased sensitivity of sensor-based measurements. One key reason for making a shift towards digital assessments is that clinical scores remain subjective in nature, and use a low resolution, ordinal scoring system. The criteria for transitioning between different scores leave much room for subjective interpretation, and cause considerable variability between and within raters in daily practice^4^.

This is exemplified by the results shown for the upper-extremity bradykinesia measure, in which we find that the measure most correlated with in-clinic MDS-UPDRS ratings - amplitude of arm-twist - is not the one that is most sensitive to change from dopaminergic medication. It is possible that while the experts are instructed to evaluate “speed, amplitude, hesitations, halts and decrementing amplitude^34^”, they may focus mostly on amplitude. Similarly, we observe a gradient of tremor measurements, both in-clinic and remotely, even within the group of participants who are rated as a 0 on the MDS-UPDRS 3.15 or 3.17. This suggests that some amount of tremor could be present, both in the clinic and at-home, even before it becomes apparent to the human eye. Indeed, it is generally a well-accepted phenomenon that tremors are easier felt or even heard (using a stethoscope) than observed by an examiner. This reinforces the need for objective sensor-based measures, and the need to evaluate these measures based on their ability to detect clinically meaningful changes rather than simply reproducing subjective clinical exams.

In people with PD, dopaminergic medication can considerably improve severity of motor signs over short time frames. This “on-off” difference is well-accepted as a clinically meaningful change, and when coupled with wearable sensors and patient-reported tagging of daily medication regimen, creates multiple “natural experiments” in the course of patients’ daily lives. These allow us to test the clinical validity^35,36^ of the PD-VME measures as pharmacodynamic/response biomarkers for people with PD in the remote setting. Indeed, we demonstrate that digital measures for tremor, upper-extremity bradykinesia and gait are able to detect significant change in patients’ motor signs before and after medication intake.

For clinical trials aiming to show disease modification, measurements that provide reliable estimates of a subject’s disease state can increase statistical power, and reduce the required sample size or trial duration. However, measuring long-term progression using infrequent measurements is difficult, because motor and non-motor signs of PD can markedly fluctuate from moment to moment^31,37^, depending on factors such as the timing of medication intake or the presence of external stressors. The increased test-retest reliability of the monthly aggregated measures from this study suggest that collecting outcome measures remotely and at an increased frequency increases their reliability, and has the potential to measure progression of the average motor sign severity.

This work is not without its limitations. The smartwatch was worn unilaterally, though PD typically exhibits asymmetrical symptom severity. Asymmetry may have particularly affected our assessments of gait which may exhibit different characteristics when observed from the most- or least-affected upper-extremity. Further analysis is needed to better understand the impact of device placement on measurement validity and reliability. Also, PD is multifaceted in nature, and signs manifest along multiple motor and non-motor domains^38^. While we present data on multiple important motor domains, additional research is needed to use the rich data collected in this study to expand this to additional motor and non-motor aspects (e.g., by using the PPG and EDA data collected). Finally, future work replicating the results presented here in a different study population is needed. In particular, a study of people with more advanced PD could enable further understanding of the impact of medication-induced dyskinesia on the sensor-based measurements. In addition to enabling exploratory analyses around non-motor symptoms, the 3-year follow-up of PPP will enable future work looking at the PD-VME’s sensitivity to long-term disease progression. The smart-watch form-factor could enable future analysis combining the PD-VME measurements together with measures obtained from passive monitoring. For signs of bradykinesia in particular, active tasks allow us to capture the intent to move, which may be difficult to capture from passively collected data.

In conclusion, these data suggest that patients engage robustly with the PD-VME, and are able to complete remote active assessments of motor function to yield data of a sufficient quality to generate digital measurements of motor signs, test their analytical validity, and assess their sensitivity to change in medication status. The system allows for an increased frequency of data collection, enabling monthly aggregation of measurements, leading to increased test-retest reliability. In turn, high reliability suggests that these measures have potential as digital biomarkers of progression. Further research is needed to more firmly establish the ability of these and other measures to serve as progression biomarkers.

## Methods

### Study design

To maximize engagement, participants were instructed to consistently wear the watch on the side that is most convenient for them. All sensor data collected in this study used a wrist-worn wearable device, the 2nd-generation of the Verily Study Watch (hardware and firmware characteristics can be found in the supplementary materials).

Inclusion criteria for this study include: PD diagnosed in the last 5 years; 18 years of age or older;

Ability to provide written informed consent; no nickel allergy (as components of the Study Watch contain this metal). Patients with co-morbidity are explicitly NOT excluded from participation. There were no inclusion/exclusion criteria based on participants’ familiarity or comfort with technology.

Sensor data is collected during the yearly in-clinic MDS-UPDRS Part III motor exams. These are conducted in both the on and off states, after overnight withdrawal of dopaminergic medication (at least 12 h after the last intake). Exams are video-recorded for quality controls and offline consensus scoring. The datasets that were used for the analysis are described in Table 1 and the Results section.

### Design of the Parkinson’s disease virtual motor exam

The PD-VME system, including participant-facing training materials, user interface, task choice and digital measures, was developed using a patient-centric approach^39^. The PD-VME comprises eight tasks designed to assess various domains of motor signs: rest and postural tremor, upper extremity bradykinesia through finger tapping, pronation-supination and repeated hand opening and closing, lower-extremity bradykinesia through foot stomping, gait and postural sway. Participant-facing instructions for each task are in Supplementary Table 1. Figure 1c shows the PD-VME user interface for the four tasks analyzed in this paper. Selection of targeted signs was informed by research on meaningful aspects of health in PD: tremor, bradykinesia and gait were identified as three of the top four symptoms that people with PD most want to improve^40^. A patient panel of PPP participants was involved throughout the design process to assess and improve the usability of the system.

During execution of PD-VME tasks, tri-axial accelerometer and gyroscope data was collected at a sample rate of 200 Hz. For each task, an initial list of concepts of interest were identified (e.g., tremor severity, quality of gait). For each concept, digital signal processing was implemented to convert the raw sensor data into 11 exploratory outcome measures (e.g., tremor acceleration, arm-swing magnitude).

### Evaluation of digital measures from the PD-VME

Participant engagement with the PD-VME, measured as the fraction of participants who performed at least one complete exam in a given week, was evaluated over the course of 70 weeks. The ability of the participants to perform the PD-VME correctly without having received in-person instructions was assessed using the assessor observations from the in-clinic PD-VME.

The analytical validity, reliability, and sensitivity to change of digital measurements from the PD-VME was evaluated. First, the analytical validity of measures, collected during the in-clinic MDS-UPDRS, was assessed using the Spearman correlation coefficient of the measure against the consensus of three independent MDS-UPDRS Part III clinical ratings. Second, the test-retest reliability in the home setting was evaluated by computing the intra-class correlation between monthly means across subsequent months for months with no missing PD-VME. Finally, the sensitivity to change was assessed by testing the ability of the remote measurements to distinguish between the off and the on states for the subset of patients in Set 2 who are on dopaminergic medication. An unsupervised PD-VME exam is determined to be in the off state if it occurred at the pre-scheduled off time *and* at least 6 h after a medication tag. Similarly, an exam is determined to be in the on state if it occurred at the pre-scheduled on time *and* between 0.5 and 4 h after a medication tag. Two measures were used to assess the magnitude of change: mean difference (and associated 95% confidence interval) and Cohen’s d. Participants taking dopamine agonists were not included in the on-off comparison because of their prolonged effect.

For each task, one outcome measure is shown in the results, selected on the basis of its high performance across all three aspects (analytical validity, test-retest reliability, and sensitivity to change) for inclusion in the results. The results of the remaining 8 measures are presented in the supplementary materials.

### Comparison of the in-clinic and remotely collected PD-VME measurements

To characterize the extent to which measures obtained from clinic-based physical exams (off) reflected patients’ signs in the remote setting (off), the distributions of participants’ in-clinic and remote PD-VME outcomes (completed within 90 days of the clinic visit) were compared. A subset of *N* = 194 participants from Set 2 who performed the PD-VME in-clinic was included in this analysis.

Figures and statistical analyses were generated using the Python programming language, using the SciPy^41^, Matplotlib^42^ and seaborn^43^ libraries. In all numerical results that follow, point estimates are followed by 95% confidence intervals in square brackets. Confidence intervals were calculated using the bootstrap method with 1000 resampling iterations.

### Reporting summary

Further information on research design is available in the Nature Research Reporting Summary linked to this article.

## Supplementary information

Supplementary materials

Reporting Summary

## Data Availability

The datasets generated during this study will be made available to qualified researchers worldwide, insofar as such requests are for analysis for which the participants in the PPP have provided informed consent. Requests for access to data are governed by a Research and Data Sharing Review Committee (RDSRC), whose role is to protect subjects’ privacy by limiting the availability of the study data and controlling access to sources of information that might potentially be used to identify the individual subjects associated with the biospecimen analyses. The RDSRC will assess the relevance and scientific quality of research proposals for which study data or material is requested^27^. Inquiries regarding access to the data can be sent to info@parkinsonopmaat.nl.

## Code availability

The code used in the study is proprietary, and will not be made available. We direct readers to the data availability section on applications for data use as a qualified researcher.
