# Author Correction: Virtual exam for Parkinson’s disease enables frequent and reliable remote measurements of motor function

**DOI:** 10.1038/s41746-022-00744-0

**Published:** 2022-12-28

**Authors:** Maximilien Burq, Erin Rainaldi, King Chung Ho, Chen Chen, Bastiaan R. Bloem, Luc J. W. Evers, Rick C. Helmich, Lance Myers, William J. Marks, Ritu Kapur

**Affiliations:** 1 Verily Life Sciences, South San Francisco, CA USA; 2 Radboud University Medical Center, Donders Institute for Brain, Cognition and Behaviour, Department of Neurology, Center of Expertise for Parkinson & Movement Disorders, Nijmegen, the Netherlands; 3 Radboud University, Institute for Computing and Information Sciences, Nijmegen, the Netherlands

**Keywords:** Biomarkers, Parkinson's disease

Correction to: *npj Digital Medicine* 10.1038/s41746-022-00607-8, published online 23 May 2022

The Code/Materials statement is amended to ‘The code used in the study is proprietary, and will not be made available. We direct readers to the data availability section on applications for data use as a qualified researcher.’ The original article has been corrected.

